# GP preferences for, access to, and use of evidence in clinical practice: a mixed-methods study

**DOI:** 10.3399/BJGPO.2023.0107

**Published:** 2023-11-29

**Authors:** Emer O’Brien, Aisling Walsh, Fiona Boland, Claire Collins, Velma Harkins, Susan M Smith, Noirin O’Herlihy, Barbara Clyne, Emma Wallace

**Affiliations:** 1 Department of General Practice, Royal College of Surgeons in Ireland (RCSI) University of Medicine and Health Sciences, Dublin, Ireland; 2 Department of Public Health and Epidemiology, RCSI University of Medicine and Health Sciences, Dublin, Ireland; 3 Data Science Centre, School of Population Health Sciences, RCSI University of Medicine and Health Sciences, Dublin, Ireland; 4 Irish College of General Practitioners, Dublin, Ireland; 5 Discipline of Public Health and Primary Care, Trinity College Dublin, Dublin, Ireland; 6 Department of General Practice, University College Cork, Cork, Ireland

**Keywords:** clinical practice guidelines, evidence-based practice, mixed methods, patient-centred care, general practice, primary health care, family practice

## Abstract

**Background:**

GPs aim to provide patient-centred care combining clinical evidence, clinical judgement, and patient priorities. Despite a recognition of the need to translate evidence to support patient care, barriers exist to the use of evidence in practice.

**Aim:**

To ascertain the needs and preferences of GPs regarding evidence-based guidance to support patient care. The study also aimed to prioritise content and optimise structure and dissemination of future evidence-based guidance.

**Design & setting:**

This was a convergent parallel mixed-methods study in collaboration with the national GP professional body in the Republic of Ireland (Irish College of General Practitioners [ICGP]). Quantitative and qualitative findings were integrated at the interpretive level.

**Method:**

A national GP survey was administered via the ICGP (December 2020) and seven GP focus groups were undertaken (April–May 2021).

**Results:**

Of 3496 GPs, a total of 509 responders (14.6%) completed the survey and 40 GP participants took part in focus groups. Prescribing updates, interpretation of test results, chronic disease management, and older person care were the preferred topics for future evidence-based guidance. GPs reported that they required rapid access to up-to-date and relevant evidence summaries online for use in clinical practice. Access to more comprehensive reviews for the purposes of continuing education and teaching was also a priority. Multimodal forms of dissemination were preferred to increase uptake of evidence in practice.

**Conclusion:**

GPs indicated that rapid access to up-to-date, summarised evidence-based resources, available from their professional organisation, is preferred. Evidence should reflect the disease burden of the population and involve multifaceted dissemination approaches.

## How this fits in

Significant literature exists outlining the barriers to the use of evidence in clinical practice. This study identified the specific requirements of GPs for rapid access to up-to-date, summarised, and evidence-based resources at the point of care. GPs preferred evidence-based guidance made available by their GP professional organisation that reflected the complex care provided in primary care. Multimodal dissemination methods and practical implementation strategies have the potential to improve uptake of this evidence in clinical practice.

## Introduction

A cornerstone of general practice is coordinating care of individuals and families.^1,2^ GPs aim to provide patient-centred care combining clinical evidence, clinical judgement, and patient priorities.^3–6^ The breadth and evolution of primary research means that this clinical evidence needs to be available in a condensed form; for example, evidence syntheses, guidelines, and policies,^7–11^ and be specific to general practice.^12^

Clinical practice guidelines, based on a comprehensive evaluation of the evidence, include recommendations intended to optimise patient care.^13^ There is a body of literature outlining the barriers to guideline implementation in clinical practice.^14–18^ A recent systematic review categorised the barriers into political and social factors (requirement of a leader to champion implementation), health system organisation factors (lack of time), guideline factors (clarity and access), health professional factors (confidence and knowledge), and patient factors (social and cultural influence).^19^ Other barriers exist such as the quantity of guidelines available and variations in the quality and the possibility of contradictory evidence.^20,21^

With up to 80% of GP consultations involving patients with multimorbidity and related polypharmacy, specific challenges exist for GPs using guidelines in practice.^22,23^ Guidelines tend to have a single disease focus, and may not provide adequate decision-support to deliver such complex care.^24–27^ A recent systematic review on barriers and facilitators to implementing guidelines in primary care identified six categories,^28^ similar to those outlined above,^19^ but also included a category on behavioural regulation; for example, incentivisation. Incorporating individual patient preferences for shared decision making is essential but can pose a challenge for GPs.^29^

Despite diverse healthcare systems and political and/or cultural differences, one of the shared central functions of many GP professional organisations is to produce evidence-based practice guidance for members in order to promote evidence-based patient care. A scoping review of international GP professional organisations demonstrated that several develop guidelines *de novo*, in collaboration with and/or endorsing other national and international guidelines.^30^ The Irish College of General Practitioners (ICGP) is the professional body for general practice in the Republic of Ireland.^31^ The ICGP Quality and Safety in Practice (QSIP) Committee coordinates the production of quick reference guides (QRGs). Some QRGs are summaries of other European clinical guidelines (including recommendations for practice), while others are evidence updates on topics where there are specific contextual issues for Irish general practice. Identifying specific interventions to improve the uptake of evidence in practice has the potential to bridge the gap between published evidence and care provision.^32–34^

### Aim and objectives

This mixed-methods study aimed to ascertain the needs and preferences of Irish GPs regarding evidence-based guidance to support patient care. It also aimed to prioritise content for future evidence-based guidance and to optimise evidence-based guidance structure and dissemination.

## Method

### Study design

This was a convergent parallel mixed-methods study involving the collection of different but complimentary data on the same area of interest.^35^ Both the quantitative survey and qualitative focus groups covered the structure, content, and presentation of ICGP QRGs, and ways to optimise their dissemination. The focus groups explored these topics in depth, whereas the survey gathered a breadth of information and covered clinical domains of current and future QRGs. Both were conducted and analysed separately. The findings were integrated at the interpretive level, using a weaving approach.^35^ This was completed by summarising the findings from both the survey and the focus groups into key points relating to the research objectives. These key points were then compared and tabulated using an assessment of fit or coherence between the findings and summarised narratively in the discussion. An assessment of fit or coherence results in confirmation, expansion, or discordance of the outcomes.^35^ The study was reported according to the Strengthening the Reporting of Observational Studies in Epidemiology (STROBE) guidelines^36^ and COnsolidated criteria for REporting Qualitative research (COREQ) guidelines.^37^

#### Survey setting, design, and analysis

Most GPs in the Republic of Ireland are private practitioners but the majority also provide care on behalf of the national public healthcare system, the Health Service Executive (HSE). This care is provided via the General Medical Service (GMS) scheme. The GMS scheme is a means-tested form of public health cover, providing medical and surgical care to the Irish population based on income and age thresholds.^38^

In 2020 the ICGP estimated its membership to be a total of 5080 members (excluding registrars). After excluding those recently retired and living abroad, the total number of active GP members in the Republic of Ireland was estimated to be 3496 doctors.^39^

A pilot survey (August 2020) was circulated to 10 academic GPs seeking feedback on ease of use, clarity, and omissions. The number of all evidence-based resources included in the survey were increased as a result of this pilot.

A cross-sectional national survey was conducted in December 2020, as part of the annual ICGP membership survey and sent to all 3496 GP members. The survey collected information on the following: 1) demographics (age and sex) and GP details (time in practice, location, and type of practice); 2) current use of all evidence-based guidance and ICGP QRGs (guidance used and reasons); and 3) future practice (preferred content, structure, and dissemination of QRGs) (see Supplementary Information S1).

The survey was administered online via SurveyMonkey. Collected data were exported to Microsoft Excel. Free-text responses (for ‘other’ and open questions) were managed by developing categories for similar responses. The data were then transferred to Stata (version 16) and descriptive statistics for each item in the survey were explored. Data were reported as percentages and frequencies.

#### Focus group setting, design, and analysis

On completion of the survey, GPs familiar with ICGP QRGs were purposively sampled and invited to participate in focus groups. Owing to low uptake, this was augmented with snowball sampling. The following were taken into consideration: sex; age; practice location (rural versus urban); and also if GPs were part of a training or teaching practice. A pilot focus group with five academic GPs was conducted. As a result of the pilot, it was decided to limit the number of participants where possible to a maximum of six, to maximise the interaction in the online setting.^40^ The focus group topic guide was developed with reference to the literature and with input from the project advisory team^41^ (see Supplementary Box S1). All focus groups were conducted online via Zoom and followed guidance on the use of Zoom for focus group interviews.^42^ Seven focus groups were conducted with a total of 40 participants. A provisional target of six focus groups was decided based on similar research^43^ and on the concept that the final number of focus groups required depends on the intersection of the research question, methods, participants, and the researchers’ interpretations of the data as an iterative process.^44,45^ On review of the demographics after six focus groups, rural GPs were under-represented; therefore, a seventh focus group with rural GPs was conducted. Each focus group lasted approximately 1 hour. Given that the questions for the focus groups were structured around distinct and pre-determined topics, this was most suited to a framework analysis approach.^46,47^

Transcription was completed by an external professional transcription company. The researcher (EOB) reviewed all the transcriptions as part of the familiarisation stage. Videos were also reviewed but group dynamics were not noteworthy, possibly owing to the online nature of the focus groups.^48–50^ The coding stage was initially completed by EOB and AW open coding two focus groups independently, using a primarily inductive approach, by generating all codes, before refining the codes after feedback from the research team. Following discussions and feedback from the research team, a set of codes and descriptions were developed and applied by EOB to the rest of the focus groups. All transcripts were transferred to the NVivo (version 12) Pro software package to complete the remaining stages. Codes were grouped into themes, and seven categories were defined and placed into the framework. This stage of development of the analytical framework was followed by indexing the transcripts, thus applying the analytical framework. The framework matrix was then created containing a summary for each category, for each focus group, for each participant, which allowed for ease of interpretation and explanations of the data.

## Results

### Quantitative results

#### Survey demographics

The survey was administered to 3496 GPs nationally with 509 responders completing it, which represented a response rate of 14.6%. A total of 175 (34.4%) responders had worked between 15 and 29 years in practice, and 336 (66.0%) spent the majority of their working week in practice. A total of 389 (76.4%) responders worked in a group practice, similar to the 2017 ICGP member’s national survey (Table 1).^51^

**Table 1.** Demographics for survey responders and focus group participants

Characteristic	Survey responders (*N* = 509), *n* (%)	Focus groups participants (*N* = 40), *n* (%)
**Age, years**		
<30	8 (1.6)	0 (0)
30–39	101 (19.8)	22 (55.0)
40–49	149 (29.3)	9 (22.5)
50–59	115 (22.6)	6 (15.0)
60–69	101 (19.8)	3 (7.5)
≥70	35 (6.9)	0 (0)
**Sex**		
Male	225 (44.2)	15 (37.5)
Female	278 (54.6)	25 (62.5)
Prefer not to say	6 (1.2)	0 (0)
**Years in practice**		
<5	61 (12.0)	14 (35.0)
5–14	110 (21.6)	16 (40.0)
15–29	175 (34.4)	5 (12.5)
>29	121 (23.8)	5 (12.5)
Retired	21 (4.1)	N/A
GP registrar	19 (3.7)	N/A
Not provided	2 (0.4)	0 (0)
**Sessions per week in practice^a^**		
1–2	16 (3.1)	2 (5.0)
3–4	37 (7.3)	7 (17.5)
5–6	86 (16.9)	11 (27.5)
7–8	148 (29.1)	14 (35.0)
>8	188 (36.9)	5 (12.5)
Not provided	34 (6.7)	0 (0)
N/A		1 (2.5)^b^
**Practice type**		
Group practice	389 (76.4)	35 (87.5)
Single-handed practice	85 (16.7)	4 (10.0)
Not provided	35 (6.9)	0
N/A		1 (2.5)^b^
**Practice location**		
Mixed	178 (35.0)	5 (12.5)
Rural	81 (15.9)	12 (30.0)
Urban	226 (44.4)	22 (55.0)
Not provided	24 (4.7)	
N/A		1 (2.5)^b^
**Teaching practice**		
Undergraduate		12 (30.0)
GP registrars		7 (17.5)
Both		8 (20.0)
None		13 (32.5)
**GP trainer**		
Yes		8 (20.0)
No		32 (80.0)

^a^General practice is organised into clinical sessions; a morning or afternoon session might be, for example, a 3-hour clinic, seeing patients every 15 minutes. ^b^A qualified GP who was not working in clinical practice at the time. NA = not applicable.

#### Survey results

##### Preferred sources of clinical practice guidance

**Figure 1.**
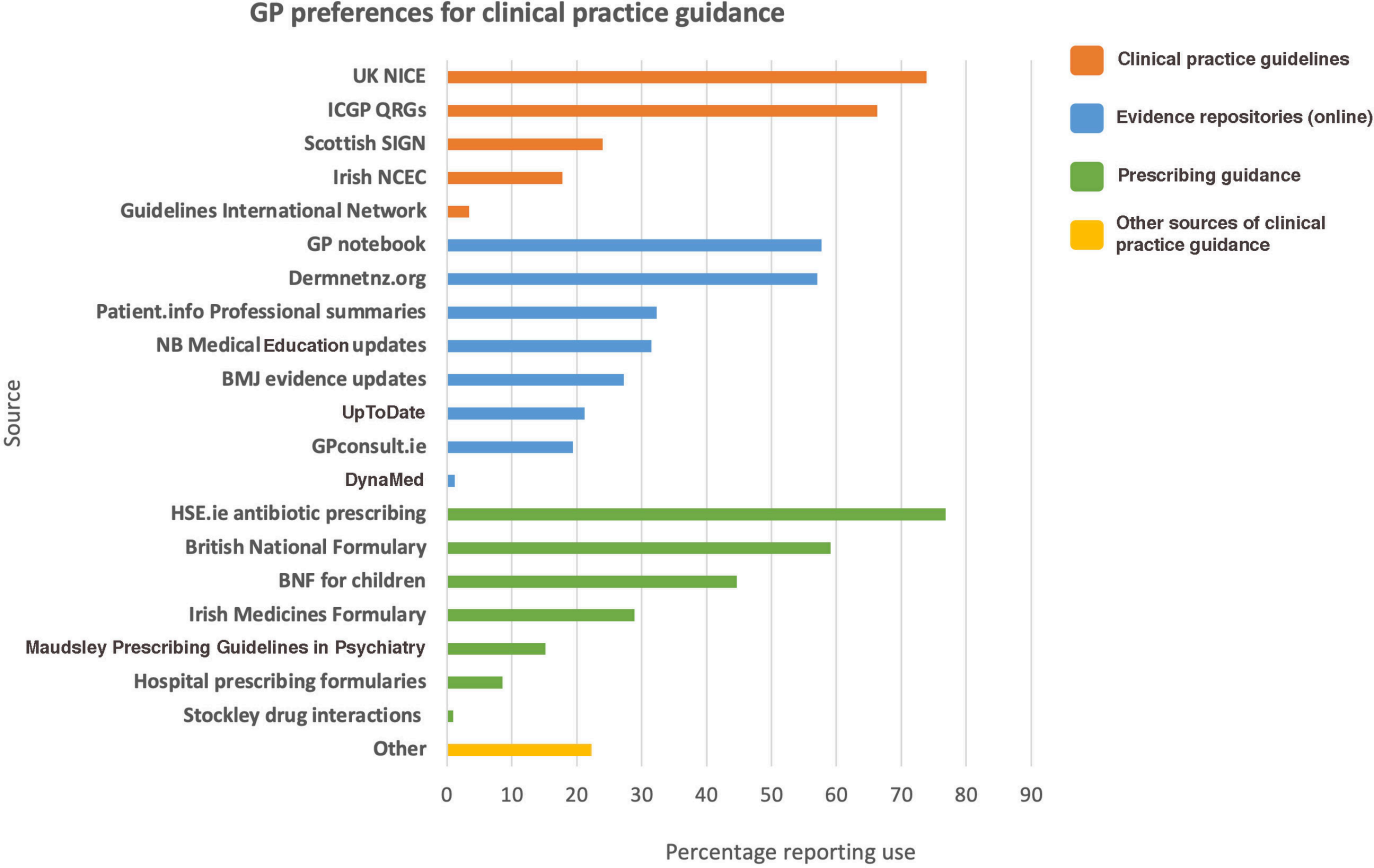
Preferred sources of clinical practice guidance (*n* = 499). BNF = *British National Formulary*. ICGP = Irish College of General Practitioners. QRG = quick reference guide. NCEC = National Clinical Effectiveness Committee. NICE = National Institute for Health and Care Excellence. SIGN = Scottish Intercollegiate Guidelines Network.

A total of 369/499 (73.9%) responders reported using the UK National Institute for Health and Care Excellence guidelines, 331 (66.3%) the ICGP QRGs, whereas only 89 (17.8%) used the Irish National Clinical Effectiveness Committee clinical guidelines. Regarding prescribing guidance, 383 (76.8%) used the Irish HSE antibiotic prescribing resource, 295 (59.1%) used the *British National Formulary*, and 144 (28.9%) used the *Irish Medicines Formulary*, a subscription medicines database specific to the Republic of Ireland. Other guidance used in practice included online evidence-based repositories such as GPnotebook (*n* = 288, 57.7%), NB Medical Education updates (*n* = 157, 31.5%), and *BMJ* evidence updates (*n* = 136, 27.3%) (Figure 1).

##### Users of ICGP QRGs

**Figure 2.**
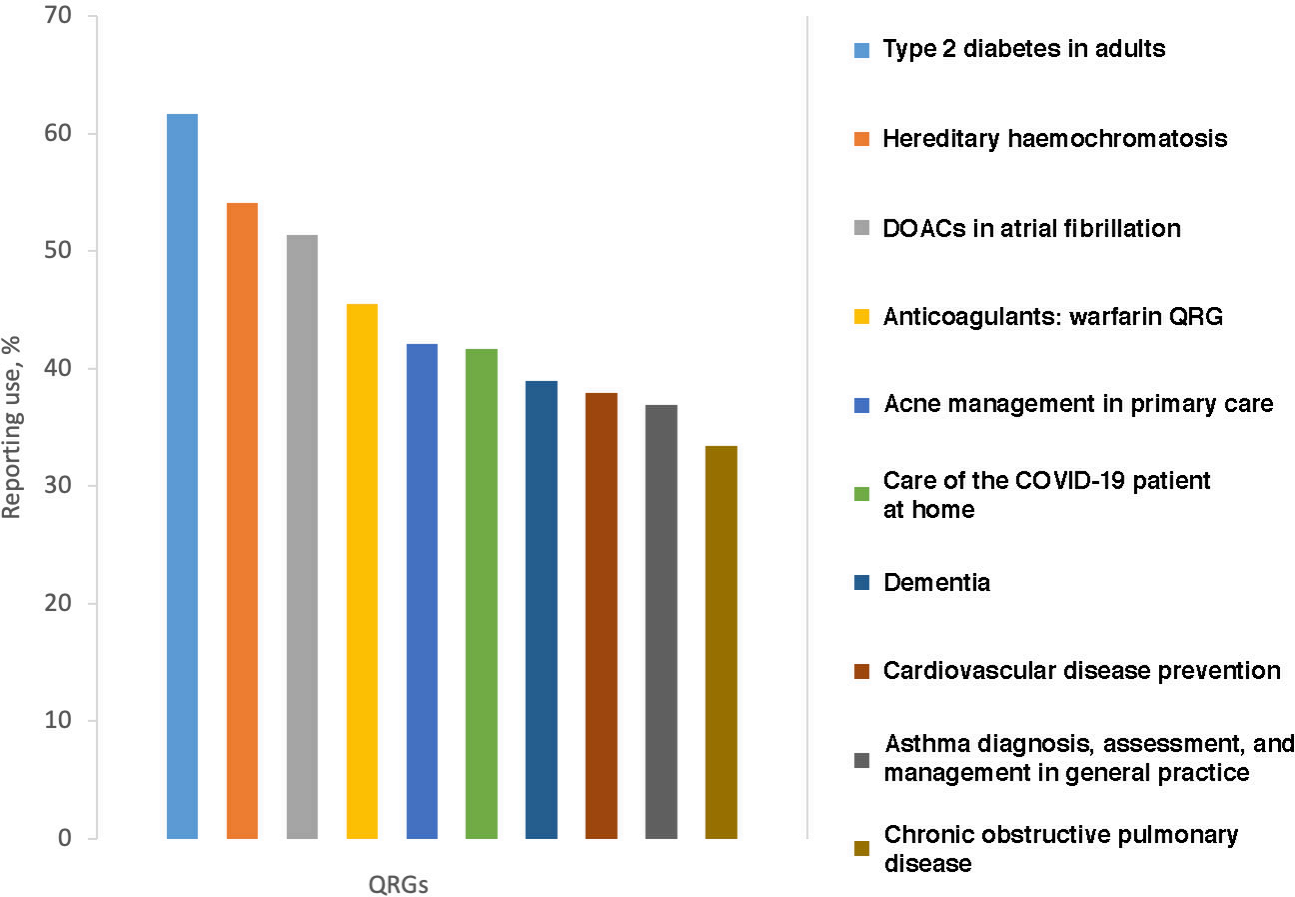
Irish College of General Practitioners top 10 QRGs used by GPs to inform patient care (*n* = 290). DOACs = direct oral anticoagulants. QRG = quick reference guide.

For the 320 (62.9%) responders who were familiar with QRGs, the most frequently used QRGs included type 2 diabetes (*n* = 179/290, 61.7%), hereditary haemochromatosis (*n* = 157/290, 54.1%), and the direct oral anticoagulants in atrial fibrillation guide (*n* = 149/290, 51.4%) (Figure 2). A list of all QRGs used to inform patient care can be found in Supplementary Table S1.

##### Future QRG content

**Figure 3.**
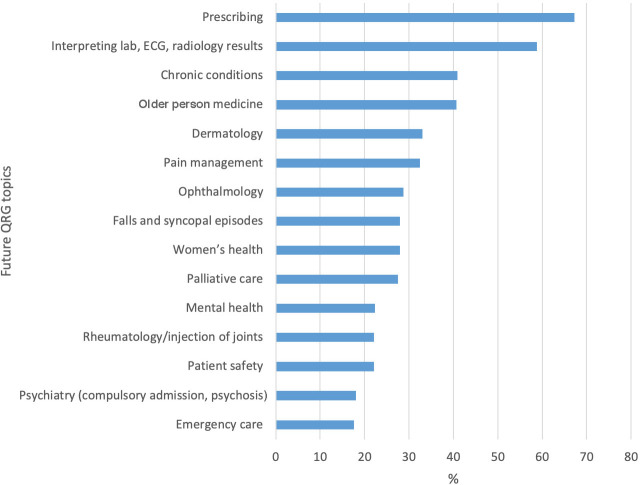
Preferences for future Irish College of General Practitioners QRGs (*n* = 403). QRG = quick reference guide.

The top clinical domains requested by survey responders for future QRG development were prescribing (*n* = 271/403, 67.2%), interpretation of test results (*n* = 237, 58.8%), management of common chronic conditions (*n* = 165, 41.0%), and older person medicine (*n* = 165, 41.0%) (Figure 3). Within the prescribing domain, specific topics of interest were drug interactions, prescribing updates, prescribing safety, and deprescribing.

##### Accessing evidence: QRGs versus all guidance documents

A total of 3/299 (1%) responders who were familiar with the QRGs accessed them every day, and 114 (38.1%) accessed them less than once a month. This is in contrast to the 220/506 (43.5%) responders who accessed any guidance resources every day. The top three reasons responders identified for accessing any guidance resources were as follows: 1) accurate, reliable, up-to-date, and trustworthy content; 2) ease of access to the resource; and 3) clear, practical, and easy-to-use structure. In comparison, those familiar with QRGs reported some difficulties in finding QRGs on the ICGP website. Other barriers to the use of QRGs were time, user-friendliness, and the length of the document. The majority of responders accessed the QRGs on their desktops, with 77 responders accessing via their phones.

##### Preferred structure and modes of dissemination

Responders (*n* = 382) indicated that future QRGs should include the following: summary document (*n* = 258, 67.5%), full document (*n* = 164, 42.9%), and infographics, educational materials, and audit ideas (*n* = 106, 27.7%), as well as patient information leaflets (PILs) (*n* = 155, 40.6%). A total of 351/385 (91.2%) responders preferred ICGP email alerts as a means for future dissemination. Other favoured future dissemination methods were webinars (*n* = 250/388, 64.4%), e-learning modules (*n* = 209/388, 53.9%), continuous medical education (CME) groups (*n* = 182/388, 46.9%), and podcasts (*n* = 77/388, 19.8%). A total of 209/386 (54.1%) responders agreed that having the QRG integrated into the general practice electronic health record (EHR) would be extremely useful.

### Qualitative findings

#### Focus group demographics

A summary of the participant demographics is presented in Table 1.

#### Qualitative findings

The framework matrix consisted of seven categories, presented in Table 2 with each of the category codes.

**Table 2.** Framework matrix showing categories and codes developed from the framework analysis

Categories	Codes
Purpose of the QRGs	Overview of a topic read outside the consultation Education — teaching (GP registrars or medical students), continued medical education, and studying for membership exams Practice audit and research QRGs need to have a dual purpose — summary and full review Specific QRGs used when returning to or starting clinical practice in Republic of Ireland and for upskilling in clinical areas with less confidence
Challenges and barriers to QRGs	QRGs do not cover all relevant topics needed for practice Lack of motivation to change from current evidence-based resources QRG a misnomer as a title Structure and format difficult to navigate Professional organisation needs to promote QRGs Not a source used in daily practice so not familiar with them Lack of time Combination of resources used in practice
Professional organisations approach to QRGs	Distribution of resources Members’ needs Accessibility challenges of the professional organisation’s website Professional organisation stature and gold standard of care
Requirements of future guidance documents	Speed of access to essential information Summary version for use in practice Standardised format Up to date and updated with changing evidence Importance of healthcare context, developed for GPs by GPs Guidance documents need key points for practice to facilitate using them in practice Need to be interactive, with, for example, frequently asked questions, hyperlinks, glossary, and visual aids (algorithms)
Dissemination of QRGs	Multimodal dissemination methods Email, webinar, podcast, video, and social media Continuous professional development meetings Application on phone Accessible and searchable online
Patient resources	Patient information leaflets, if limited resources then should focus on QRGs
Implementation of guidance documents	Practical strategies International network Quality improvement initiatives Use of the electronic health record

QRG = quick reference guide.

### Purpose of the QRGs

Most participants concurred that QRGs provide a comprehensive overview of a topic for use by GPs outside the consultation. In their current format, participants felt they had limited use within a consultation. This was in contrast to the expectation that QRGs should provide quick access to relevant information, and for this reason other sources (Figure 2) were more commonly used in practice. There was a suggestion that the QRGs could serve a dual purpose by creating a summary for use in practice and the full review for education.

Participants discussed the circumstances in which they have used QRGs (Table 2). For example, one participant who trained abroad and was now practising in the Republic of Ireland found the coeliac disease and haemochromatosis QRG particularly useful as these conditions are more prevalent in the Republic of Ireland. Some participants discussed reading QRGs in clinical areas that they lack confidence in; for example, one participant found the eating disorders QRG particularly useful to read after dealing with a patient presenting with this condition:

*‘… what bloods to do, does she need a DEXA* [Dual Energy Xray Absorptiometry]*, what can I do in the meantime. I felt a lot more equipped on following up with the results after reading it* [QRG].*‘* (Focus group [FG]1: participant [P]1)

QRGs were also used as education tools for teaching and learning. GPs involved in both undergraduate and postgraduate GP training referred to them as the gold standard for teaching, and GP registrars used them when revising for their professional membership examination. Other examples included using the medication review QRG to support the completion of a practice audit on prescribing, and use of the coeliac disease QRG during a CME group to inform decisions regarding changes to local practice.

### Challenges and barriers to the QRGs

Participants spoke about challenges and barriers to using QRGs. The PDF format and length of QRGs was a noticeable barrier, as it requires time to navigate and identify pertinent information. Most participants used other online resources in practice that are designed to provide rapid accessible answers to clinical questions, aided by hyperlinks and visual aids.

As time is a considerable challenge in their working life, most participants are working with a list of priorities, therefore spending time reading QRGs ultimately gets deferred:

*‘I find when the resources are there you always put it off. There’s always something else to do first. So it is great to have them, but it’s just finding the time to get round to it.’* (Focus group pilot:P1)

According to most participants the title ‘*quick reference guide*’ is a misnomer. What they expect is a one-page summary; however, what they find is a full review of the topic, which during a busy day can be a source of frustration:

*‘... when you say quick reference guide, I’m thinking, oh it’s like the BMJ infographic. I’m going to get it all, I’m going to get it on a page, my life is going to be saved. And then I click into it and it’s ninety-two pages long.’* (FG3:P1)

### Professional organisation’s approach to QRGs

All participants agreed that the central role of the GP professional organisation should be in responding to the needs of its target audience. Most participants expressed a preference for evidence guidance produced by their professional organisation. Guidance from the professional organisation is considered to be trustworthy and provides the gold standard of care, based on the Irish context. This trust that the guidance is aimed at the local population provides confidence when using the evidence in patient care, not provided by competing resources. An example was provided by a single-handed rural GP. They described the need to be proficient in the management of resistant hypertension (for example, prescribing a third antihypertensive) owing to the long waiting time for cardiology outpatients in their area of practice. This example was used to discuss the need for the organisation to be aware of the context of care and to respond to these challenges.

### Requirements of future guidance documents

All participants expressed the importance of improving QRGs for use in practice for busy working GPs. All participants concurred that speed of access to essential information is key. There was a general consensus among most participants that there is a need for a summary document for each QRG:

*‘Really what you need is that very quick access to bullet point information, particular points that you need to manage a particular consultation.’* (FG1:P4)

Most participants suggested the use of visual aids to speed up interaction with QRGs and to save valuable practice time:

*‘... if every illness and treatment plan came in a little algorithm I would be the happiest doctor on the planet.’* (FG5:P5)

The need for QRGs to be up to date was discussed by most participants. Concerns were expressed by some that it is not clear if QRGs are updated as evidence changes, or whether updates are made on periodic review, meaning some information can be out of date:

*‘... they may lag slightly, you mightn’t be getting the most up-to-date information just because the review isn’t yet pencilled in.’* (FG4:P5)

### Dissemination of QRGs

Multiple modes of dissemination were discussed by participants, with a consensus that whatever the dissemination method (Table 2), rapid access to information is a priority. Emailing was highlighted with a suggestion that the use of concise key points within the email, including links to the QRG, might improve engagement. There were mixed opinions about the use of an app as a way of disseminating the QRGs, with some participants suggesting that having the information available on their phone via an app would be useful for home visits and out-of-hours practice. However, others expressed concerns about downloading another app that may not be used.

### Patient resources

Participants focused on patient information leaflets (PILs) in particular, and most were content with the current PILs they were using in practice. Concerns were expressed by some participants that if the professional organisation focused on developing PILs, that this might divert resources away from the QRGs.

### Implementation of guidance documents

There was extended discussion about the considerable challenges of working in general practice currently and how it would be necessary to outline how a GP working in the Irish healthcare system should implement best practice. One participant suggested developing a network for guideline production and/or adaptation, bringing together guideline-producing bodies both nationally and internationally. There were reservations about integrating the QRG into EHRs as a template for use during the consultation and the potential time burden associated with this approach.

## Discussion

### Summary

On integration of the survey and focus group findings, expansion occurred when comparing the key findings from both (Table 3). Although there were some differences in demographics between the focus groups and survey participants, this did not appear to influence the consistency of findings between the different samples. GPs identified the need for rapid access to up-to-date, pertinent, and relevant clinical information for use in clinical practice. Management of type 2 diabetes was the QRG most commonly reviewed by Irish GPs. Evidence updates being developed by their own professional organisation were seen by study participants as the gold standard of care and having access to full reviews for education purposes was a priority. Leading topics identified for future QRGs were prescribing, interpretation of test results, chronic disease management, and older person care. The importance of the content being relevant to the complex care delivered by GPs in the context of their healthcare system was emphasised. Time-poor GPs required rapid access to online, searchable, summary documents with visual aids, with multimodal methods of dissemination preferred. Use of the EHR for implementation of guidance documents was favoured in the survey but not in the focus groups owing to reservations about changing the purpose of the QRG if it was integrated as an EHR template for completion during the consultation. Their professional organisation leading on practical implementation strategies was critical to GPs, as they faced challenges of providing evidence-based, patient-centred care in a distinct clinical environment (see Table 3).

**Table 3.** Integration of the quantitative and qualitative findings comparing summary key points from the survey and focus groups, using an assessment of fit

Research objectives	Quantitative findings	Qualitative findings	Integration outcome
Needs and preferences for evidence-based guidance to support patient care	Guidelines Guidance documents Evidence repositories Prescribing tools Type 2 Diabetes top QRG used to inform patient care	Accurate, rapid, reliable, up-to-date, evidence-based, concise, and clinically relevant information For use in practice and as an education tool Professional organisation-published evidence updates accepted as the gold standard for quality care provision Key points for practice	Expansion
Content for future guidance documents	Prescribing updates Interpretation of results Management of common chronic conditions Older person care	Relevant to the complex care provided in primary care In the context of the healthcare system	Confirmation and expansion
Structure of future guidance documents	Summary document Full document Infographics Educational material Patient information leaflets	Hyperlinks or search box Visual aids Standardised format Up to date Ease of access to clinically relevant information	Confirmation and expansion
Dissemination of guidance documents	Email alerts Webinars eLearning modules CME groups Podcasts Implementation of guidance documents by integrating them into the EHR	Multimodal methods Available online Speed of access to online content, searchable Reservations about integrating QRGs into the EHR as a questionnaire-based template to promote implementation of the guidance document	Confirmation and expansion Dissonance
		Practical implementation strategies, for example, through local CME groups International GP professional organisation network — for the development of evidence updates Quality improvement initiatives	Expansion

CME = continuous medical education. EHR = electronic health record. QRG = quick reference guide.

### Strengths and limitations

This national survey is representative of Irish GPs, with ICGP members accounting for >85% of practising GPs in the Republic of Ireland. The low survey response rate is in line with previous ICGP surveys^51^ and is a feature of surveys involving healthcare professionals.^52^ Time constraints and survey burden are recognised barriers but the use of personalised contact and incentives may have improved the response rate.^52^

The main researcher, a GP themselves, remained focused on their positionality while conducting the focus groups. It was challenging to maintain impartiality and listen actively to the points discussed. This ‘insider’ role was balanced and maintained by >1 qualitative analyst and in seeking feedback from the research team from different professional backgrounds.

### Comparison with existing literature

Participants in this study expressed a preference for evidence-based guidance that focuses on issues related to prescribing, management of common chronic conditions, and older person care. This preference reflected the areas of increased need in the population and the increased complexity of general practice care.^53^ A recent UK study predicted that between 2015 and 2035, the proportion of adults aged >35 years living with ≥4 chronic conditions will double.^54^ Prescribing for patients with multimorbidity is complex,^55^ and with increasing numbers of medicines comes increased risk including potentially inappropriate prescribing, where the balance of medication risks outweigh benefits for the individual.^56^ The present study has highlighted that GPs require appropriate guidance to support this complex clinical care.^26,57^

Speed of access to online, up-to-date summaries and relevant information at the point of care were highlighted as important for GPs who participated in this study. This is consistent with other studies where the main barrier to guideline use identified is relevance to general practice. Other barriers were ease of access, length, and complexity of the guideline document.^28^ A recent scoping review reported that GP professional organisation-led clinical guidelines vary greatly in length, and usability was likely impacted by reliance on downloadable PDFs available from the organisation’s website.^30^

### Implications for practice

The findings of this study have emphasised the role of GP professional organisations to not only provide access to evidence-based guidance, but also to provide leadership and an understanding of the tacit knowledge that influences a GP’s practice. Most GPs have developed ‘mindlines’, described as ‘*collectively reinforced, internalised tacit guidelines*’,^58^ developed over time and influenced by personal experiences, the healthcare system, and their community and patients.

Informed by the findings of this study, a new format has been developed for QRGs published by the ICGP. This novel format has been utilised with the recent publication of a deprescribing in general practice QRG. This addresses GP feedback regarding their preferences for how evidence is presented and disseminated. A content prioritisation exercise for future evidence-based guidance has been completed based on the knowledge gaps identified by GPs in the survey and will be used to prioritise future topics.

It is important to have evidence-based guidance underpinning service provision and to align changes in national health policy with guidance development. For example, in 2015 as part of the GP chronic disease management programme for diabetes,^59^ both the model of care and the clinical guidance was based on the updated ICGP QRG on the diagnosis and management of uncomplicated type 2 diabetes in adults. There is clear potential for a more formal coordinated approach to identifying guidelines for development based on the disease burden of the primary care population and local policy.

The authors believe this study has international implications as similar challenges exist in primary care worldwide.^28,30^ There is clear potential for collaboration between GP professional organisations internationally, to share resources, reduce duplication of effort, and promote standardisation in the development and implementation of evidence-based guidance.

## Data

Additional data can be found at the Open Science Framework: https://osf.io/a75k3/?view_only=16a872182aa04b9da8f8e3e58c1f54cf
